# Successful sequencing of the first SARS-CoV-2 genomes from Croatian patients

**DOI:** 10.3325/cmj.2020.61.302

**Published:** 2020-06

**Authors:** Igor Jurak, Tomislav Rukavina, Oliver Vugrek

**Affiliations:** 1 Department of Biotechnology, University of Rijeka, Rijeka, Croatia; 2 Faculty of Medicine, University of Rijeka and Teaching Institute of Public Health, Rijeka, Croatia; 3 Laboratory for Advanced Genomics, Ruđer Bošković Institute, Zagreb, Croatia *ovugrek@irb.hr*

Since the outbreak of atypical pneumonia cases in the Wuhan region, China, in late 2019, the virus causing what is now called COVID-19 has rapidly spread around the globe. As of late May 2020, more than 5.5 million people have been infected and almost 350 000 have died. The first case of infection in Croatia was identified on February 25, 2020 (*https://www.hzjz.hr/priopcenja-mediji/covid-19-priopcenje-prvog-slucaja*/), and the current number of confirmed infected patients is 2244, with 100 deaths. The National Civil Protection Directorate promptly implemented strict measures to limit the virus transmission, which have decreased the number of daily new cases from almost 100 at the peak of the epidemic to below 10. Bringing the epidemic under control, which means that the basic reproduction number (R_0_) is below 1, transmission is controlled, and there is an ample number of beds and respirators available in hospitals, created the conditions for the restrictions to be eased. Although this has brought relief to the population and the hard-hit economy, it is impossible to predict further developments beyond the devastating effect on the economy. The efforts of countless laboratories around the world have resulted in an unprecedented accumulation of knowledge on severe acute respiratory syndrome coronavirus 2 (SARS-CoV-2). Yet, many aspects of the virus biology, transmission, and treatment remain elusive.

Responding to the urgent need to contribute to global efforts in understanding COVID-19 and the virus transmission, epidemiologists, molecular biologists, and virologists from Zagreb and Rijeka have teamed up and initiated a virus genome sequencing project. Despite the general country lockdown, restricted travel, and a devastating one-in-a-100-years earthquake that hit Zagreb on March 22, we have successfully sequenced the first SARS-CoV-2 genomes circulating in Croatia.

Briefly, we obtained 33 RNAs from the Institute of Public Health in Rijeka collected as part of routine diagnostics between March 22 and April 10, 2020. In all samples, SARS-CoV-2 had been confirmed by RT-qPCR (generously provided by Neven Sučić) before sequencing. The samples of adequate quality (21 distinct viral RNA isolates) were used for next-generation-sequencing (NGS) on an Illumina platform (San Diego, CA, USA) at Ruđer Bošković Institute. Our NGS approach was based on an in-house designed panel, consisting of 173 amplicons, covering the complete SARS-CoV-2 genome. We obtained high-quality whole genome sequences for seven samples, with average sequence coverage between 6000 and 12 000 × . The sequences were instantly deposited in GISAID, the main SARS-CoV-2 genomes open-source database (*https://www.epicov.org*). In addition, we retrieved partial sequences, covering 50% to 84% of the viral genome, for the remaining 14 samples, which can be used for partial genomic analyses. The main question is what the virus genome sequences can be used for. There are several important applications.

First, although not directly a scientific matter, it was important to show that Croatia has technical expertise and capacity to successfully sequence the virus and contribute to global efforts. This success is not limited only to SARS-Cov-2 research, as the platform can be extended to monitor the seasonal circulating flu viruses, measles outbreaks, West Nile virus, etc.

Second, by comparing our virus sequences with those of other countries, we can conclude on the virus origin and, possibly, the number of entries, which is of high public and epidemiological interest. From the virus outbreak in Wuhan till its arrival to Croatia, SARS-CoV-2 mutated several times, and currently there are at least three distinct branches (clades) carrying specific mutations. These are named clade S (ORF 8, amino acid exchange L84S), G (S protein, D614G), and V (NS3, amino G251V). Six of our genomes belong to the clade G, and one genome is still under investigation. Nonetheless, our preliminary results indicate multiple virus entries and, as expected, close similarity to the viruses sequenced in Italy and Austria (1).

Third, there are many reasons to further monitor the virus mutation dynamics. For example, some virus regions mutate at a significantly higher rate than others, indicating a strong selective pressure. Indeed, the greatest number of mutations has been identified within the S gene, the main viral antigen. This is relevant for the selection of antigens or novel drug targets in vaccine and treatment development, respectively. The virus could also possibly show a population-specific adaption, which is immensely interesting but has to be confirmed by large virus genome studies (2). Tracing the mutation dynamics can be applied to evaluate restriction measures. For example, during the COVID-19 pandemic, there have been reports of frequent infection of medical workers, which can significantly limit the health care capacities. Thus, understanding if in-hospital infections were caused by a patient-medical worker transmission or a breach in safety controls (bringing virus by infected medical workers from elsewhere) might help improve the implemented measures. Furthermore, sequencing techniques can be applied to evaluate travel restrictions (3).

Forth, the COVID-19 pandemic is fraught with contradictions. On one hand, older age and chronic/pre-existing medical conditions are well-documented risks for the disease severity, and on the other, some younger, previously healthy, individuals develop severe illness and even die. In addition, a significant number of individuals infected with SARS-CoV-2 with mild symptoms or no symptoms at all were identified – ie, seemingly healthy people may inadvertently spread the disease. Such puzzling contradictions might be explained by differences in the virulence and pathogenicity of the circulating strains, and thus, it is mandatory to delve into the core of the viral genome to the last nucleotide to confidently answer these questions. Indeed, recently, Yao et al have investigated a number of patient-derived isolates *in vitro*, showing that some mutations might impact the virulence of SARS-CoV-2 (4).

In summary, the COVID-19 pandemic is currently waning in many European countries, including Croatia. Hopefully, the rest of the world will follow the same trend. Many lessons have been learned, and by far more wait to be addressed. Thus, a thorough analysis of global measures to mitigate the pandemic will hopefully help to create advanced procedures to tackle future challenges. We strongly believe that our effort in designing the sequencing platform will contribute to a better preparedness for a possible second SARS-CoV-2 wave or a similar disaster.

Acknowledgment We thank Filip Rokić, Lovro Trgovec-Greif, and Neven Sučić for the preparation of samples and sequencing analysis, and overall contribution to the project.
